# Different Thermal Treatment Methods and TGase Addition Affect Gel Quality and Flavour Characteristics of *Decapterus maruadsi* Surimi Products

**DOI:** 10.3390/foods11010066

**Published:** 2021-12-28

**Authors:** Qiang Li, Shumin Yi, Wei Wang, Yongxia Xu, Hongbo Mi, Xuepeng Li, Jianrong Li

**Affiliations:** 1 College of Food Science and Technology, Bohai University, Jinzhou 121013, China; lq00742021@163.com (Q.L.); wwll812002@163.com (W.W.); xuyx1009@126.com (Y.X.); mihongbo1001@163.com (H.M.); xuepengli8234@163.com (X.L.); lijr6491@163.com (J.L.); 2 National & Local Joint Engineering Research Center of Storage, Processing and Safety Control Technology for Fresh Agricultural and Aquatic Products, Jinzhou 121013, China; 3 National R&D Branch Center of Surimi and Surimi Products Processing, National and Local United Engineering Lab of Marine Functional Food, Jinzhou 121013, China

**Keywords:** *Decapterus maruadsi*, surimi product, thermal treatment method, gel quality, flavor characteristics

## Abstract

*Decapterus maruadsi* surimi products were prepared using the thermal treatment methods of boiling (BOI), steaming (STE), back-pressure sterilization (BAC), roasting (ROA), microwaving (MIC), and frying (FRI), respectively. The effect of glutamine transaminase (TGase) addition was also investigated. The moisture distribution, water retention, microstructure, color, fracture constant, protein secondary structure, chemical forces, and flavor components of each sample were determined. The differences in gel and favor characteristics between *D. maruadsi* surimi products caused by thermal treatment methods were analyzed. The results showed that BOI, STE, and FRI had the largest protein secondary structure transitions and formed dense gel structures with high fracture constant. The kinds of flavour components in BOI and STE were completer and more balanced. The high temperature treatment available at BAC and FRI (110 °C and 150 °C) accelerated the chemical reaction involved in flavor formation, which highlighted the flavor profiles dominated by furans or esters. The open thermal treatment environments of ROA, MIC, and FRI gave them a low moisture content and water loss. This allowed the MIC to underheat during the heat treatment, which formed a loose gel structure with a low fracture coefficient. The addition of TGase enhances the gel quality, most noticeably in the ROA. The aldehyde content of the FRI was enhanced in the flavor characteristic. The effect of adding TGase to enhance the quality of the gel is most evident in ROA. It also substantially increased the content of aldehydes in FRI. In conclusion, different heat treatments could change the gel characteristics of surimi products and provide different flavor profiles. The gel quality of BOI and STE was consistently better in all aspects.

## 1. Introduction

Surimi is the minced meat obtained from the processing of raw fish [1], and it can be considered as concentrated myofibrillar protein [2]. Marine fish such as pollock are currently used for surimi production. However, the scarcity of marine resources and the fluctuating price of surimi have led to a shortage of raw materials for surimi production. The production of surimi is gradually shifting to the use of lower value marine and freshwater fish [3]. Japanese scad (*Decapterus maruadsi*) belongs to the Carangidae family of Perciformes and is widely distributed in East Asian waters. The global annual catch is 5 million tons, ranking third among all fish species [4,5]. The use of *D. maruadsi* in food processing is currently focused on the protein functional properties and functional peptide extraction [6,7]. Relatively little research has been performed on its use in minced fish and minced fish products.

Due to its high protein content, surimi has strong gel properties after heating [8]. The most commonly used thermal treatment method is water-bath heating, which produces relatively satisfactory and stable gelation quality and is the traditional method of producing surimi [9]. The traditional method of processing surimi products is mainly in the form of heat transfer. With the development of technology, many new food processing technologies have been used to develop surimi products such as back-pressure sterilization [10], microwave heating [11], and ohmic heating [12]. However, there are differences in texture and flavor between surimi products prepared by different processing methods. Microwave radiation has a different effect on the aggregation behavior of myosin compared to conventional water bath heating, where the alternating electric field induced by microwave heating is able to produce a tearing effect that is more favorable to the unfolding of myosin molecules at higher temperatures [13]. By combining microwave treatment with conventional water bath treatment, replacing the second water-bath step with microwave treatment, the gel quality of the obtained surimi products could be further improved [11]. In the treatment of surimi products using air frying, it was found that the surface water content sharply decreased with increasing temperature, and the hardness, viscosity, and chewiness of the fried surimi increased. When the temperature exceeded 160 °C, the oil degraded, and peroxidation occurred, and new flavor compounds such as aldehydes and ketones were produced [14]. Prolonged heating of surimi at 90 °C accelerates the aggregation of proteins with unchanged structure and reduces hydrophobic bonding, thereby weakening hydrophobic interactions and reducing the ability of proteins to bind aldehydes [15].

Gel characteristics are one of the most important properties of surimi products, mainly in terms of gel strength and the ability to retain moisture. It has been found that the gelation properties of surimi products can be effectively improved by adding exogenous edible substances. For example, some polysaccharides are able to bond with proteins through hydrogen bonds, and then they absorb water and swell to fill and support the gel mix, thus enhancing the gelation properties of surimi [16]. The addition of glutamine transaminase (TGase) catalyzes the formation of ε-(γ-Gln)-Lys bonds between the lysine ε-amino group and the γ-carboxy amide group on glutamine, allowing more cross-linking of myosin and increasing the gel strength and water retention of surimi products [17,18].

In this study, we compared the effects of different heating methods on the gel quality and flavor characteristics of *D. maruadsi* surimi products, and analyzed them in terms of changes in protein secondary structure and chemical forces. The role of TGase addition on these effects was also analyzed. It is expected that BOI and STE may have the best gel characteristics and flavor characteristics among surimi products prepared by various thermal treatment methods. In *D. maruadsi* surimi products with a higher heat treatment temperature, individual flavor substances may generate a large amount of such as FRI. The aim is to provide a theoretical reference for the production of surimi products with different quality characteristics through different treatment methods in actual production.

## 2. Materials and Methods

### 2.1. Materials

First, ice-fresh *D. maruadsi* about 300 g size, which was caught in winter and purchased from Chengyang Market in Qingdao, China, was used. TGase was used with an enzymatic activity of 1 million U, and was produced by Dongsheng Biotechnology Co., Ltd., in Taixing, China. Compound phosphate was produced by Enmiao Food Co., Ltd., in Henan, China.

### 2.2. Preparation of D. maruadsi Surimi

After removing the scales, head, internal organs, and abdomen of chilled *D. maruadsi*, they were cut into fillets. After the fish fillets were cleaned, the meat was harvested by a meat picker and then finely filtered. The finely filtered surimi was rinsed with 0 °C purified water at 5 times the volume for 15 min, and then dehydrated at 1200 rpm·min^−1^ for 15 min. The rinse and dehydration were performed 3 times, and 0.15% salt was added during the third rinse. Finally, 4% sucrose and 4% sorbitol were added to the dehydrated surimi, which was evenly mixed. After being divided, it was vacuum-sealed and stored at −80 °C for later use. This *D. maruadsi* surimi was analyzed and found to contain 76.73% water, 11.00% protein, 2.52% fat, 1.86% ash, and 7.89% carbohydrates.

### 2.3. Preparation of D. maruadsi Surimi Products

The *D. maruadsi* surimi was thawed, weighed, and chopped for 3 min, and then 2.5% salt was added to the mixture, which was chopped for 3 min. The remainder of the additives (NT: 0.5% phosphate complex; WT: 0.5% phosphate complex, and 0.5% TGase) were then added, and the meat was chopped for 3 min. All chopping was carried out at 0–10 °C and −0.6 bar. The chopped mixture was then poured into a 32 mm diameter nylon casing to produce fish sausages 11–12 cm in length. The process for preparing *D. maruadsi* surimi products using different thermal treatment methods is shown in Table 1.

Each of the above thermal treatment methods includes NT and WT, a total of 12 treatment groups; each treatment group prepared a total of 7 sausages. The prepared sausages were stable for 12 h at 4 °C.

### 2.4. Determination of Moisture Retention

#### 2.4.1. Moisture Content

The moisture contents of *D. maruadsi* surimi products were determined in triplicate according to the method proposed by the Association of Official Analytical Chemists [19]. 3 parallel samples per set.

#### 2.4.2. Low-Field Nuclear Magnetic Resonance (NMR)

The samples were processed in the same manner as that used for hydrogen proton distribution imaging. The transverse relaxation time T2 of the samples was measured using a Carr–Purcell–Meiboom–Gill (CPMG) pulse sequence with a low-field NMR instrument (Niumag Electric Co., Shanghai, China). Parameter settings: SFI (MHz) = 22, P90 (μs) = 14, SW (KHz) = 100, Echocnt = 8000, NS = 4, τ (μs) = 200, P3 (μs) = 80, TR (μs) = 800, RG1 (db) = 20, RG2 = 3, and 3 parallel samples per set.

#### 2.4.3. Water Loss Rate

The method used was slightly modified based on Lv et al. [20]. The surimi gel samples were cut into thin slices with a mass of 1.3–1.5 g and a thickness of 2–3 mm. The surface water was gently wiped off the slices with filter paper, and then the slices were accurately weighed as *M*_1_. The slices were then wrapped with 3 layers of dry filter paper and placed in a 50 mL centrifuge tube, centrifuged at 4 °C and 5000× *g* for 15 min, then immediately removed and weighed as *M*_2_. Three parallel sets of samples were used for each group. The water loss rate was calculated using the formula.

$$\mathrm{Water}\;\mathrm{loss}\;\mathrm{rate}\;(\%)=\frac{M_{1}-M_{2}}{M_{1}}\times 100$$

### 2.5. Determination of Gel Properties

#### 2.5.1. Microstructure

The samples were cut into cubes with a side length of 8 mm and frozen at −80 °C for 30 min. The samples were cut into thin slices of 6 μm thickness using a frozen sectioning machine, placed on slides, stained with the hematoxylin-eosin kit, and placed under an ortho-optical microscope (80-i, Nikon, Tokyo, Japan) for observation.

#### 2.5.2. Whiteness

The samples were first placed at room temperature for 30 min and then cut into cylinders 24 mm in height with neat, smooth, and crack-free surfaces. The luminance (L*), red-green (a*), and yellow-blue (b*) values of the samples were determined using a colorimeter. The whiteness values of the samples were calculated by the following equation [21]. Three parallel samples were used for experimentation.

$$\mathrm{Whiteness}=100\;-\sqrt{{\left(100-L*\right)}^{2}+{a*}^{2}+{b*}^{2}}$$

#### 2.5.3. Puncture Test

The puncture test employed a texture analyzer (TA.XT. PLUS, Stable Micro System, UK) using a slight modification of the method by Wijayanti et al. [22]. Prior to measurement, samples were left at room temperature for 30 min and then cut into cubes with a side length of 2.5 cm. Five analyses were carried out using a P/5S model probe (spherical plunger 5 mm in diameter) with a pre-test and post-test velocity of 1 mm·s^−1^, a compression shape of 50%, and a trigger force of 15 g. The measured fracture force and fracture deformation were used to calculate the fracture constant [23].

### 2.6. Protein Conformation and Force Changes

#### 2.6.1. Raman Spectroscopy

Raman spectroscopy was performed with a slight modification according to the method of Poowakanjana et al. [24]. The samples were cut into 10 mm × 10 mm × 2 mm slices and placed on slides. A high-resolution Raman spectrometer was used with a semiconductor laser as the excitation source, laser wavelength 532 nm, power 100%, acquisition time 30 s, two scans, spectral resolution 0.6 cm^−1^, spatial resolution 400 nm, and Raman shift range 400–3600 cm^−1^. The percentage of α-helixes, β-sheets, β-turns, and irregularly coiled structural units of proteins was calculated using the method of Alix et al. [25]. 3 parallel samples per set.

#### 2.6.2. Chemical Forces

The method used was slightly modified from that of Tan et al. [26]. First, 3 g of chopped sample was weighed in a 50 mL centrifuge tube. Then, 15 mL of SA (0.6 mol·L^−1^ NaCl) was added, and the solution was homogenized at 8000 r·min^−1^ for 3 min, cooled at 4 °C for 1 h, centrifuged for 10 min (4 °C, 10,000× *g*), and the supernatant was then stored at 4 °C. Next, 15 mL of SB (1.5 mol·L^−1^ urea, 0.6 mol·L^−1^ NaCl mixture) was added to the SA-treated precipitate, and the procedure as described above for SA was repeated. Subsequently, 15 mL of SC was added (8.0 mol·L^−1^ urea, 0.6 mol·L^−1^ NaCl) to the SB-treated precipitate, and the procedure as described above for SA was repeated. For the last addition, 15 mL of SD (8.0 mol·L^−1^ urea, 0.6 mol·L^−1^ NaCl, 0.5 mol·L^−1^ β-mercaptothion) extract was added to the SC-treated precipitate, and the procedure as described above for SA was repeated. The concentration of the supernatant was then determined using the biuret method. The concentration of protein dissolved in SA was contributed by ionic bonds, the concentration of protein dissolved in SB was contributed by hydrogen bonds, the concentration of protein dissolved in SC was contributed by hydrophobic interactions, and the concentration of protein dissolved in SD was contributed by disulfide bonds. 3 parallel samples per set.

### 2.7. Volatile Flavor Substances

Volatile compounds from *D. maruadsi* surimi products were identified by headspace-gas chromatography-ion mobility spectrometry (HS-GC-IMS) (FlavourSpecR, Dortmund, Germany) using a slight modification of the method by Li et al. [27]. First, 2 g of sample was placed in a 20 mL headspace syringe and incubated in a stirrer at 60 °C (500 rpm for 15 min). Into a heated syringe (65 °C), 500 μL of headspace was then injected using a heated syringe. Pre-separation was accomplished by gas chromatography equipped with an MXT-5 capillary column (15 m, 0.53 mm) at 60 °C. The flow rate of the carrier gas nitrogen (99.99% purity) was 2 mL·min^−1^ for 2 min and 150 mL·min^−1^ for 20 min. The analytes were then ionized in an IMS ionization chamber (45 °C) and further separated. Analysis was carried out using the resulting difference maps, fingerprinting, and principal component analysis (PCA) maps.

With n-ketone compounds C4–C9 as an external reference, the retention index (RI) of each compound was calculated by an automated mass spectral deconvolution and identification system. The identification of volatile compounds uses RI and drift time to compare with the GC-IMS library, and the content of volatile compounds is quantitatively analyzed based on the peak intensity of HS-GC-IMS. 3 parallel samples per set.

### 2.8. Statistical Analysis

All graphs presented in this study were prepared using Origin 9.0, and all data were statistically analyzed using SPSS 19.0 software. The data were subjected to one-way analysis of variance (ANOVA) and bivariate correlation analysis and are expressed as mean ± SD. Differences in measurements were considered significant for *p* < 0.05.

## 3. Results and Discussion

### 3.1. Analysis of Moisture Retention

**Figure 1:**
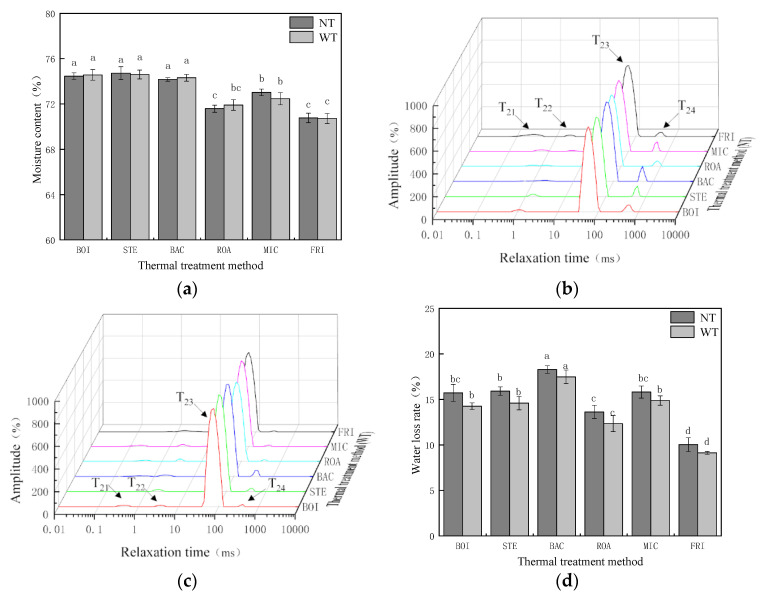
Effect of different thermal treatment methods and the addition of TGase on the moisture retention of *D. maruadsi* surimi products. (**a**): Moisture content; (**b**): moisture distribution status (NT); (**c**): moisture distribution status (WT); (**d**): water loss rate. BOI: boiled *D. maruadsi* surimi products; STE: steamed *D. maruadsi* surimi products; BAC: back-pressure sterilized *D. maruadsi* surimi products; ROA: roasted *D. maruadsi* surimi products; MIC: microwaved *D. maruadsi* surimi products; FRI: fried *D. maruadsi* surimi products; NT: group of without TGase; WT: group of with TGase. Completely different letters in the same indicator indicate significant differences between the same columns (*p* < 0.05).

The moisture contents of the *D. maruadsi* surimi products were affected by the different thermal treatment methods but not by the addition of TGase. As shown in Figure 1a, there was a higher (*p* < 0.05) and a similar moisture content (74.17% to 74.72%) for the boiled (BOI), steamed (STE), and back-pressure sterilized (BAC) *D. maruadsi* surimi products. This was due to the fact that the BOI, STE, and BAC were wrapped in the nylon casings during the thermal treatment process, which inhibited the evaporation of water from the surimi products, resulting in relatively high and similar (*p* > 0.05) moisture content in these three groups. Conversely, the roasted (ROA), microwaved (MIC), and fried (FRI) *D. maruadsi* surimi products were not wrapped in the casings, and there was a significantly (*p* < 0.05) lower moisture content than the samples in the other three treatment groups due to the evaporation of water. The moisture content of the MIC was moderate (NT: 73.03%, WT: 72.46%), while the lowest moisture content was for ROA and FRI in that order (NT: 71.58%, 71.91%; WT: 70.77%, 70.72%). The degree of moisture evaporation in the surimi products without the casing was influenced by the heating rate [28], which was related to the heating temperature and time. In a comparative study of surimi prepared by microwave and steam heat treatment methods, it was also found that the higher heating rate for the microwave treatment group resulted in a relatively low moisture content [29]. The heating time for the ROA was longer (18 min), while the highest temperature was recorded in the FRI (150 °C); therefore, the moisture content in the ROA and FRI was more similar (*p* > 0.05) and significantly lower than the other four groups (*p* < 0.05).

Figure 1b,c shows the spectra obtained by low-field nuclear magnetic resonance technology of *D.maruadsi* surimi products prepared by different heat treatment methods. The T_2_ relaxation time is related to the binding force and degree of freedom of the hydrogen protons and reflects the chemical environment of the hydrogen protons in the sample. The more bound hydrogen protons or the smaller the degree of freedom, the shorter the T_2_ relaxation time, and the more to the left of the T_2_ spectrum peak [30]. The water molecules corresponding to the T_21_ (0–1 ms) and the T_22_ (1–17 ms) bind to the food through strong hydrogen bonds, called bound water, which is the water that binds most closely to the food components [30]. The T_23_ (20–235 ms) is formed by immobile water, which is tightly combined with single water molecules [30]. The T_24_ (>200 ms) reflects free water, which is weakly bound to food [30]. Table 2 lists the peak area of each spectral peak, and the side reflects the content of each kind of moisture.

**Figure 2:**
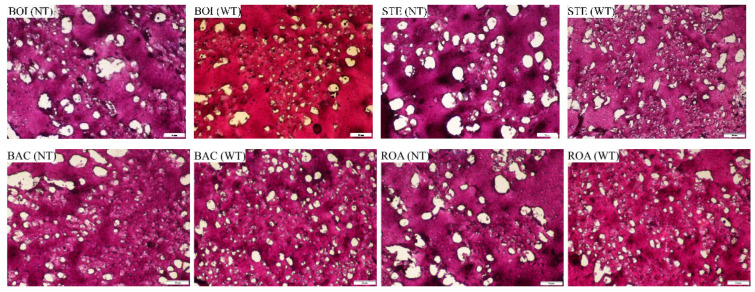

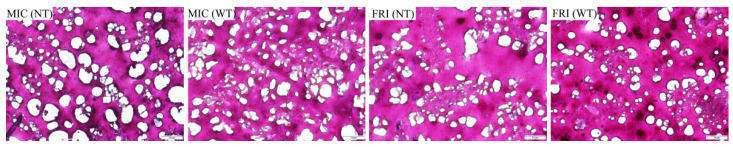
Effect of different thermal treatment methods and the addition of TGase on the microstructure of *D. maruadsi* surimi products. BOI: boiled *D. maruadsi* surimi products; STE: steamed *D. maruadsi* surimi products; BAC: back-pressure sterilized *D. maruadsi* surimi products; ROA: roasted *D. maruadsi* surimi products; MIC: microwaved *D. maruadsi* surimi products; FRI: fried *D. maruadsi* surimi products; NT: group of without TGase; WT: group of with TGase.

Figure 1d expresses the effect of different thermal treatment methods and TGase addition on the water loss rate of *D. maruadsi* surimi products. The water loss rate of surimi product is usually influenced by the combined interaction with their water distribution state and gel structure [31,32]. The effects of the different thermal treatment methods on the bound water in the *D. maruadsi* surimi products were not significant (*p* > 0.05) with (WT) or without TGase (NT) addition. Therefore, the trend of water loss between samples in each treatment group remained the same, both in NT and WT. However, the addition of TGase changed the water distribution status of each sample in WT. The T_24_ peak area decreased from 27.66–65.24 to 3.79–34.45, and the T_23_ peak area increased from 851.85–954.77 to 954.91–1113.34 in each treatment group. This may be due to the fact that TGase induces the cross-linking of proteins, and the dense gel structure retains more water, thus prompting a shift from free water to immobile water [33]. This directly caused their water loss rate in the WT to be reduced. The thermal treatment conditions for the BOI and STE were relatively similar; therefore, the differences in the free and immobile water content of the samples in both groups were not significant (*p* > 0.05), and the immobile water content was higher in all of the four other groups. However, the content of free water was higher compared to the other treatment groups. This also directly resulted in the water loss rates of BOI and STE being at an intermediate level among the treatment groups. The free water content in the BAC was significantly higher (*p* < 0.05) than that of the samples from the other treatment groups. This made FRI have the highest (*p* < 0.05) water loss rate (NT: 18.29%; WT: 17.48%), which was caused by the higher content of free water due to the looser gel structure. Zhang et al. also made the same discovery when preparing Alaska pollock surimi gels using back pressure sterilization equipment [34]. The water loss rate of ROA, MIC, and FRI and their water distribution state have a relationship consistent with the above analysis content. However, it should be noted that the change in water distribution caused by the surimi gel structure is not the only determinant of the water loss rate. Figure 1a has shown that there are differences in moisture content among the samples of each treatment group, which is also one of the reasons that affect the water loss rate. For example, the water loss rate of FRI was significantly lower than that of the samples from the other treatment groups, probably due to the combined effect of its dense gel structure (Figure 2), the high temperature (150 °C), and open heat treatment environment resulting in significant evaporation of water. For this reason, the water loss rate, moisture content, T_21_ + T_22_ peak area, T_23_ peak area, and T_24_ peak area of each treatment group were analyzed by correlation (Table 3) to verify.

It can be seen from Table 3 that when TGase was not added (NT), the water loss of the samples in each treatment group was significantly and positively correlated with their free water content and moisture content (*p* < 0.05). Even with the addition of TGase (WT), the water loss rate was still significantly and positively correlated (*p* < 0.05) with the free water content. This is due to the fact that TGase facilitates the conversion of free water to less mobile water [33], as illustrated by the positively correlated (*p* < 0.05) between water content and less mobile water content in WT. In summary, the water loss of the samples in each treatment group is influenced by the change in moisture content due to the change in free water content. Therefore, it is not possible to evaluate the effect of heat treatment on the gel quality of surimi products based on changes in water loss alone. Further experiments are required to verify this. For example, the free water content of MIC was significantly (*p* < 0.05) lower than that of BOI and STE, but the water loss rate was not significantly different, conjecturing that the gel of MIC might be poorer.

### 3.2. Analysis of Gel Properties

#### 3.2.1. Microstructure

Figure 2 shows microscopic views of the microstructure of the *D. maruadsi* surimi products prepared by different thermal treatment methods. The distribution and presence of water in the gel can be seen based on the observed pores in the surimi gel [35]. The addition of TGase was effective in reducing the pores in the *D. maruadsi* surimi products and making their distribution more uniform. This suggests that the addition of TGase enhanced the water retention of *D. maruadsi* surimi products while improving their gel structure [36]. The size and distribution of pores in the boiled (BOI) and steamed (STE) *D. maruadsi* surimi products were relatively similar. The pores of the back-pressure sterilized *D. maruadsi* surimi products (BAC) were unevenly distributed and different sizes, which resulted in a higher water loss rate. It is speculated that this may be due to the short low-temperature gelation time of BAC and the relatively long residence time in the gel deterioration temperature band, which allows for insufficient protein structure unfolding and deeper protein hydrolysis, ultimately resulting in a poorer gel structure. The pores in the roasted *D. maruadsi* surimi products (ROA) were less numerous and more evenly distributed. This may be due to the decrease in its water content, which increased the relative protein content of the *D. maruadsi* surimi products and resulted in the formation of a tighter gel structure after heat treatment [37]. Similarly, in the fried *D. maruadsi* surimi products (FRI), which had the lowest moisture content, not only were there fewer pores evenly distributed, but the area of the pores also decreased. However, the microwaved *D. maruadsi* surimi products (MIC) with the same decreased water content had a larger pore area. This result verifies the conjecture of MIC’s water retention capacity and gel structure in the previous analysis.

The results of previous studies have all shown that the microwave heating method is effective in improving gel properties such as water retention of surimi gels [11,32]. However, the gel structure of MIC in this study was loose and had poor water retention. This may be due to the fact that the heating rate of microwaves depends on the dielectric properties of the material, and the dielectric properties of mixed surimi are determined by the type and content of the material [13,38]. Moisture is the component found in the highest content and with a large dielectric constant; therefore, its content can directly affect the dielectric properties of surimi products. Some studies have shown that at a microwave frequency of 2450 MHz, the dielectric constant and dielectric loss factor of fish sausage decrease as the moisture content (80%, 78%, 76%) decreases [29]. The initial water content of the surimi gel in this study was relatively low (75%), which resulted in the material underheating during microwave heating and developing a loose gel structure with low water retention capacity.

#### 3.2.2. Whiteness

The water content, water retention, and the structural properties of surimi product were all able to influence its color [39,40]. The effects of different thermal treatment methods and the addition of TGase on the color of *D. maruadsi* surimi products are shown in Table 4. The addition of TGase significantly increased the brightness and whiteness of the samples in each treatment group. This may be due to the ability of TGase to promote the cross-linking of myosin heavy-chains, resulting in a tighter and flatter structure of the surimi gel and enhancing its reflectance to light, while other compounds (maltodextrin) in commercial TGase powder can also cause light scattering effects [17,41]. Whether in the group of without (NT) or with (WT) TGase, boiled (BOI) and steamed (STE) *D. maruadsi* surimi products also had similar whiteness values due to their similar heat treatment temperatures (*p* > 0.05). BOI and STE had the highest whiteness values compared to the other treatment group samples, due to their higher moisture content and dense gel structure, which reflects light better. The moisture content of back-pressure sterilized *D. maruadsi* surimi products (BAC) were similar to that of BOI and STE. However, due to its shorter low-temperature gelation time and longer residence time in the gel degradation temperature band, the gel structure was loosened, thereby reducing the light reflection effect, so the whiteness value was low. In addition, the yellow-green value (b*) of the BAC was significantly higher than that of the other treated samples, indicating their overall yellow color. Some studies have shown that the color of surimi products gradually changes from white to pale yellow after high temperature and pressure treatment [42]. According to the qualitative analysis results of volatile compounds in samples of each treatment group in this study, it was found that the detection intensity of furan flavor substances in BAC was significantly higher (*p* < 0.05) than samples of other treatment groups. It is speculated that the darker color of BAC may be due to the more violent Maillard reaction during the heat treatment process, which in turn produces some pigment-like substances. The whiteness values for roasted (ROA) and fried (FRI) *D. maruadsi* surimi products were relatively similar (*p* > 0.05) and were at an intermediate level in all treatment groups. It is speculated that this is because ROA and FRI are both heated in an open environment, and the heating temperature was high, which greatly reduces the water content and increases the protein concentration and finally forms a dense gel structure. However, the decrease in water content relatively weakens its reflection of light, so the whiteness and L* are lower than BOI and STE. Although microwaved *D. maruadsi* surimi products (MIC) were also heat-treated in a relatively open environment, its heating principle is different from ROA and FRI. Heat transfer is used for roasting and frying, whereas in microwaving, the conversion of energy (microwave energy) to heat takes place [43]. Additionally, the amount of moisture determines how much microwave energy is absorbed by the surimi product [29]. The resulting insufficient temperature rise makes the gel structure of the MIC looser. Therefore, the overall reflection of MIC to light was poor, so the whiteness value was low.

#### 3.2.3. Puncture Tests

**Figure 3:**
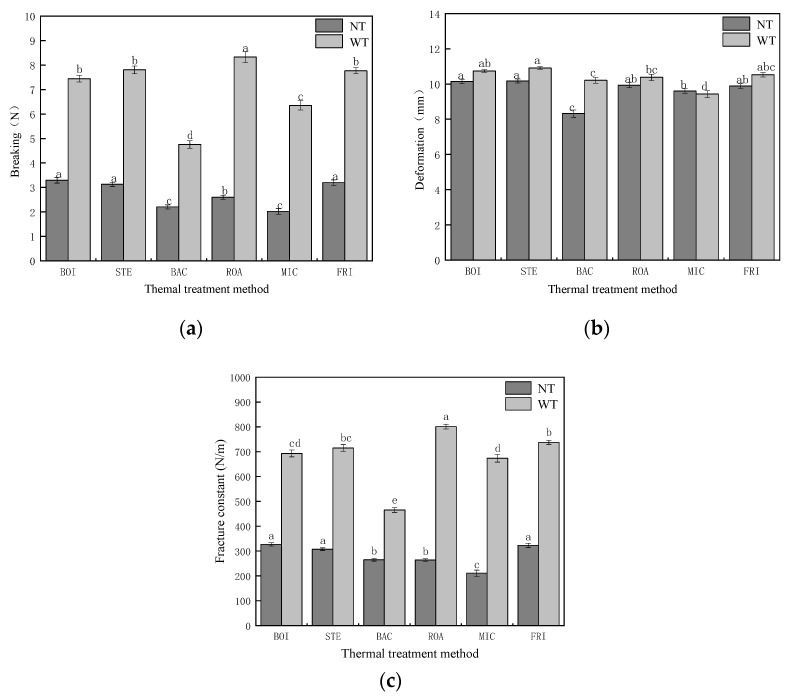
Effect of different thermal treatment methods and the addition of TGase on the fracture constant of *D. maruadsi* surimi products. (**a**): Breaking; (**b**): deformation; (**c**): fracture constant. BOI: boiled *D. maruadsi* surimi products; STE: steamed *D. maruadsi* surimi products; BAC: back-pressure sterilized *D. maruadsi* surimi products; ROA: roasted *D. maruadsi* surimi products; MIC: microwaved *D. maruadsi* surimi products; FRI: fried *D. maruadsi* surimi products; NT: group of without TGase; WT: group of with TGase. Completely different letters in the same indicator indicate significant differences between the same columns (*p* < 0.05).

The effects of different thermal treatment methods and the addition of TGase to the fracture constant of *D. maruadsi* surimi products are shown in Figure 3. The addition of TGase was effective in increasing the fracture constant of the *D. maruadsi* surimi products prepared by each thermal treatment method from 210.92–326.83 N·m^−1^ to 465.15–801.20 N·m^−1^. This is due to the ability of TGase to catalyze the generation of ε-(γ-Gln)-Lys bonds between lysine ε-amino groups and glutamine γ-carboxyamido groups, with more myosin heavy chain proteins cross-linking and forming covalent bonds to promote gel formation [17,18].

The breaking forces (3.29, 3.13, 3.20 N) and fracture constants (326.83, 307.50, 322.92 N·m^−1^) of boiled (BOI), steamed (STE), and fried (FRI) *D. maruadsi* surimi products were relatively similar (*p* > 0.05) when TGase was not added, and all were significantly higher (*p* < 0.05) than the values of the samples from the other treatment groups. It is assumed that the higher fracture constants of BOI and STE are due to the denser and more homogeneous gel structure, and the ability of the water in the surimi gel to be uniformly dispersed in it, providing a more thorough filling effect [44]. Although the filling effect of water in FRI was less, the lower water content led to an increase in the relative concentration of protein, which in turn led to a more compact and rigid structure in *D. maruadsi* surimi products [45,46]. The breaking force and fracture constants of back-pressure sterilized *D. maruadsi* surimi products (BAC) were low (225.03 g, 264.85 N·m^−1^). This was because the high temperature and pressure treatment conditions made the fragile gel structure of *D. maruadsi* surimi products, reducing both the breaking force and the deformation [34]. Numerous studies have shown that microwave heating can effectively enhance the fracture constant of surimi products [11,32]. However, the lower fracture constant (210.92 N·m^−1^) of microwaved *D. maruadsi* surimi products (MIC) in this study could be attributed to the lower moisture content of the surimi mixture, resulting in a lower dielectric constant and dielectric loss factor, which did not allow for satisfactory warming during microwave heating. This is consistent with the analysis results of the poor water retention of the MIC.

When TGase was added, the fracture coefficients (693.16, 715.28, 737.59 N·m^−1^) of BOI, STE, and FRI remained high. The breaking force (849.70 g) and the fracture constant (801.20 N·m^−1^) of roasted *D. maruadsi* surimi products (ROA) were more significantly increased. This may be due to the fact that the relative concentrations of TGase and its interacting proteins are increased as water evaporates during heating, enhancing the cross-linking effect of TGase on myosin heavy chains [47]. Although the heating environment of FRI was similar to that of ROA, the addition of TGase enhanced its fracture constant slightly less than that of the ROA. This may be due to the higher heating temperature (150 °C) of the FRI, which resulted in a decrease in the enzymatic activity of TGase [48]. Therefore, the increase in the FIR fracture coefficient by TGase was not as great as that of ROA.

### 3.3. Analysis of Protein Conformation and Force

#### 3.3.1. Analysis of Protein Secondary Structure

**Figure 4:**
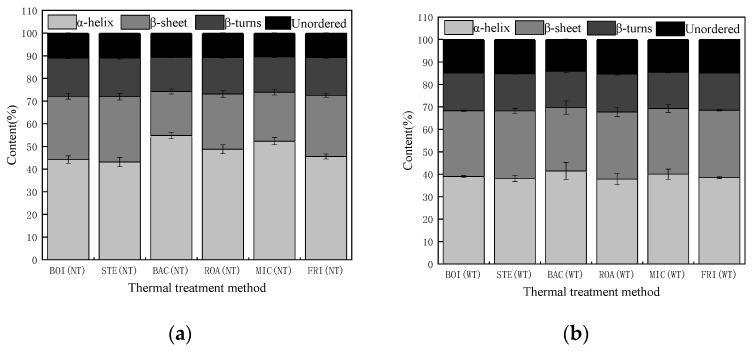
Effect of different thermal treatment methods and the addition of TGase on the protein conformation of *D. maruadsi* surimi products. (**a**): NT; (**b**): WT. BOI: boiled *D. maruadsi* surimi products; STE: steamed *D. maruadsi* surimi products; BAC: back-pressure sterilized *D. maruadsi* surimi products; ROA: roasted *D. maruadsi* surimi products; MIC: microwaved *D. maruadsi* surimi products; FRI: fried *D. maruadsi* surimi products; NT: group of without Tgase; WT: group of with Tgase.

The effect of different thermal treatment methods on the protein secondary structure of *D. maruadsi* surimi products is shown in Figure 4. Without the addition of TGase, the α-helix content in the proteins in boiled (BOI), boiled (STE), and fried (FRI) *D. maruadsi* surimi products were relatively similar (*p* > 0.05), and all were at lower levels, 44.20%, 43.12%, and 45.57%, respectively. This suggested that a more complete unfolding of the α-helix of BOI, STE, and FRI occurred. The unfolded part of the α-helix structure may exist in the system in the form of a peptide chain, which provides a basis for the formation of the β-sheet structure in the subsequent heating process [49,50]. Therefore, the content of β-sheet structures in BOI, STE, and FRI was also similar and the greatest (27.93%, 28.87%, 26.99%).

Although the compositions of the protein secondary structures in BOI, STE, and FRI were relatively similar, there should be differences in the processes of change. Studies have shown that as the thermal treatment temperature increases, the α-helix content gradually decreases [51]. Therefore, it is speculated that BOI and STE have the advantage in time during the transition from α-helix structure to β-sheet structure, while FRI has an advantage in temperature. Back-pressure sterilized *D. maruadsi* surimi products (BAC) exhibited the highest content of α-helixes (54.83%), probably because the high temperature and pressure conditions of the back-pressure sterilization treatment disrupted structures such as unordered protein and β-sheets, allowing the relative content of α-helixes to be elevated [34]. The content of α-helixes in roasted *D. maruadsi* surimi products (ROA) (48.75%) was slightly higher than that of BOI, STE, and FRI, and the content of β-sheets was 24.39%.

It is hypothesized that the reason for this may be due to the use of air as the heating medium during the roasting stage, which resulted in a low heating rate. The content of α-helixes in microwaved *D. maruadsi* surimi products (MIC) (52.32%) was only lower than that of BAC, and such a result is consistent with the analysis of higher water loss in MIC. There was no significant difference in the content of β-turns and unordered protein in the samples from each treatment group (*p* > 0.05).

The addition of TGase reduced the difference in the protein secondary structure of the *D. maruadsi* surimi products in each treatment group, but the overall trend did not change. Compared to the absence of TGase, the α-helix of the protein was reduced, and the unordered content was increased in all treatment groups. This may have occurred because TGase catalyzed the formation of more unordered protein connected by ε-(γ-Gln)-Lys bonds [35,52]. ROA exhibited the greatest increase in unordered content compared to the absence of TGase addition, with a boost of approximately 42.53%. This is consistent with the significant increase in the ROA fracture constant after adding TGase. This may be due to the fact that the heating medium for the roasting treatment is air, which has a slower rate of heat transfer. This results in a slower rate of temperature increase in the center of the surimi products, and thus, a slower rate of reduction in TGase activity [53].

#### 3.3.2. Analysis of Chemical Forces

**Figure 5:**
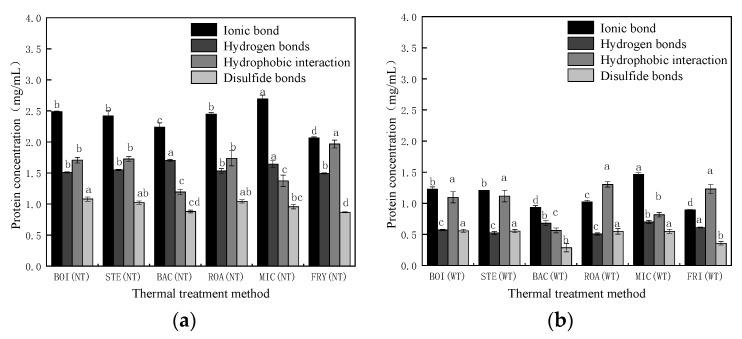
Effect of different thermal treatment methods and the addition of TGase on the chemical action of *D. maruadsi* surimi products. (**a**): NT; (**b**): WT. BOI: boiled *D. maruadsi* surimi products; STE: steamed *D. maruadsi* surimi products; BAC: back-pressure sterilized *D. maruadsi* surimi products; ROA: roasted *D. maruadsi* surimi products; MIC: microwaved *D. maruadsi* surimi products; FRI: fried *D. maruadsi* surimi products; NT: group of without TGase; WT: group of with TGase. Completely different letters in the same indicator indicate significant differences between the same columns (*p* < 0.05).

The influence of different thermal treatment methods on the chemical forces in *D. maruadsi* surimi products is shown in Figure 5. When TGase was not added (NT), the composition of the chemical force was similar in boiled (BOI), steamed (STE), and roasted (ROA) *D. maruadsi* surimi products. This may have occurred because the heating temperature and time of the three sets of samples were relatively close. There were low levels of ionic bonds, hydrophobic interactions, and disulfide bonds in back-pressure sterilized *D. maruadsi* surimi products (BAC), which were 2.24, 1.19, and 0.28 mg·mL^−1^, respectively. Back-pressure sterilization treatment was able to decrease the content of ionic bonds and hydrophobic interactions in surimi products, possibly because electrostatic interactions in ionic bonds can be involved in the aggregation of myosin molecule tails [54,55]. Additionally, the high-temperature conditions of the back-pressure sterilization treatment disrupted the intermolecular electrostatic interactions and promoted the unfolding of myosin tails, while also inhibiting the re-aggregation of myosin tails. This resulted in a poorer three-dimensional protein meshwork and degraded gel properties.

The lower number of hydrophobic interactions of BAC may be due to the faster heating rate, which allowed the exposed hydrophobic groups to be encapsulated again. Due to insufficient heating temperature, the destruction of ionic bonds and hydrogen bonds in microwaved *D. maruadsi* surimi products (MIC) were relatively low, and its content was high, at 2.69 and 1.64 mg·mL^−1^, respectively. Fried *D. maruadsi* surimi products (FRI) exhibited the highest content of hydrophobic interactions (1.97 mg·mL^−1^) and the lowest content of disulfide bonds (0.27 mg·mL^−1^). The formation of disulfide bonds in myofibrillar protein gels mainly occurs in the low-temperature treatment stage. When the temperature continues to rise (>60 °C), the content of disulfide bonds gradually decreases and is negatively correlated with the increase in temperature [56].

The addition of TGase (WT) reduced the content of ionic bonds, hydrogen bonds, hydrophobic interactions, and disulfide bonds in the samples of each treatment group. This may be due to the fact that TGase induced the cross-linking of proteins to form polymers that inhibited the exposure of hydrophobic residues and sulfhydryl groups in myofibrillar protein, resulting in a decrease in hydrophobic interaction and disulfide bond content. The decrease in ionic bonds and hydrogen bonds may be caused by the cross-linking effect of TGase on the protein, which changes the spatial structure of the protein. TGase improves the gel properties of *D. maruadsi* surimi products mainly by the formation of non-disulfide covalent bonds [52]. The addition of TGase resulted in a relatively small reduction in hydrophobic interactions of ROA. This may be due to the combined effect of heating temperature, heating time, and changes in the moisture content of the roasting treatment.

The correlation between the fracture constant and protein secondary structure of *D. maruadsi* surimi products prepared by different thermal treatment methods and the correlation with chemical forces are shown in Table 5. In the NT, the fracture constants for *D. maruadsi* surimi products were highly significant and negatively correlated with α-helix content (*p* < 0.01), with a highly significant positive correlation with β-fold and β-turn content (*p* < 0.01), and a significant positive correlation with unordered content (*p* < 0.05). When TGase was added, the fracture constant for *D. maruadsi* surimi products was highly significantly and positively correlated with unordered content (*p* < 0.01) due to the generation of a large number of irregular curl structures maintained by non-disulfide covalent bonds. The fracture constant for *D. maruadsi* surimi products was negatively correlated with ionic bond content, significantly and negatively correlated with the hydrogen bond content (*p* < 0.05), significantly and positively correlated with hydrophobic interactions (*p* < 0.05), and positively correlated with the disulfide bond content (*p* > 0.05). When TGase was added (WT), the correlation between the fracture constant and ionic, hydrogen, and disulfide bonds did not change for the *D. maruadsi* surimi products. However, the positive correlation with hydrophobic interactions became highly significant (*p* < 0.01), which was related to the substantial increase in the fracture constant of ROA by TGase and the higher level of hydrophobic interaction content.

Due to the different heating conditions for each treatment group, the influence on the content of each chemical force was quite different, and thus, the fracture constants for *D. maruadsi* surimi products prepared by different heating methods were not completely determined by a single chemical force. This leads to a smaller degree of correlation between fracture constant and a single chemical force. However, the contents of hydrogen bonds and hydrophobic interactions were the main forces affecting the fracture constant of the six *D. maruadsi* surimi products, with hydrophobic interactions being the main force maintaining the gel conformation.

### 3.4. Qualitative Analysis of Volatile Flavor Substances

**Figure 6:**
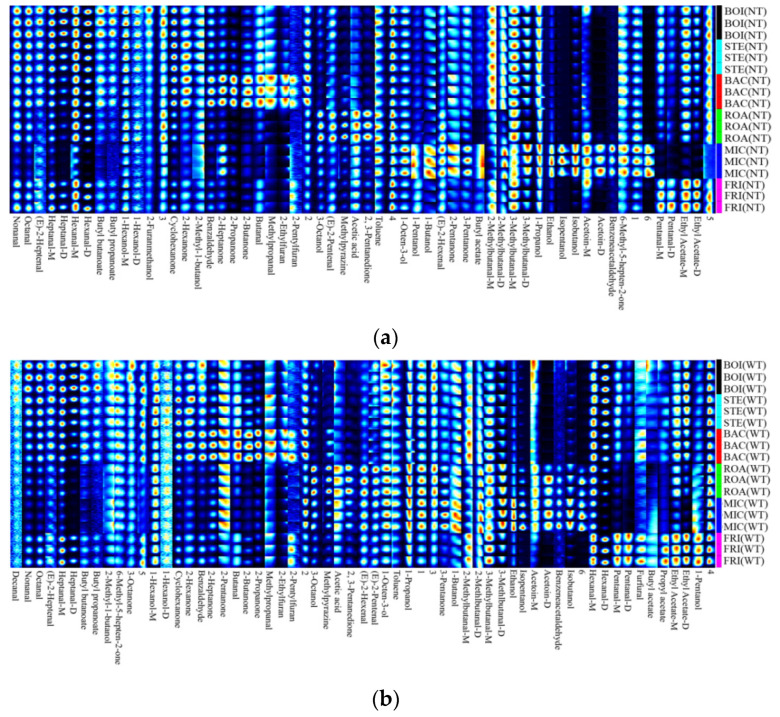
The analysis of volatile flavor substances by fingerprint map of surimi products subjected to different thermal treatment methods. (**a**): NT; (**b**): WT; BOI: boiled *D. maruadsi* surimi products; STE: steamed *D. maruadsi* surimi products; BAC: back-pressure sterilized *D. maruadsi* surimi products; ROA: roasted *D. maruadsi* surimi products; MIC: microwaved *D. maruadsi* surimi products; FRI: fried *D. maruadsi* surimi products; NT: group of without TGase; WT: group of with TGase.

The fingerprints of the volatile substances of the minced *D. maruadsi* surimi products prepared by different thermal treatment methods are shown in Figure 6. The specific detection intensity of each volatile compound can be viewed in the Supplementary Materials.

As shown in Figure 6a, a total of 44 volatile compounds were detected in NT, of which 14 are aldehydes. It has been reported that aldehydes are the main flavor components in surimi products [57,58]. Nonanal, hexanal, heptanal, and valeraldehyde are usually produced by the oxidation of unsaturated fatty acids such as linoleic acid and linolenic acid [59]. Nonanal, hexanal, and heptanal are considered to be the main sources of fishy odor [60], valeraldehyde has a cheese flavor [61], and 2/3-methylbutanal has a fried smell [57]. In addition, the Maillard reaction process also forms aldehydes such as butyraldehyde [62]. Nonanal, heptanal, hexanal, 2/3-methylbutanal, and butanal were detected with high intensity and were strongly represented in both boiled (BOI) and Steamed (STE) *D. maruadsi* surimi products. Luo et al. [57] also found similar findings when they studied the difference in flavor characteristics of surimi products after reheating by microwave, boiling, steaming, and frying. As shown in Figure 6b, a total of 47 volatiles was detected in WT, including 16 aldehydes. Nonanal, heptanal, hexanal, pentanal, 2-methylbutanal, and 3-methylbutanal were detected with high intensity and were strongly represented in both fried *D. maruadsi* surimi products (FRI) and STE, while most demonstrated weak intensity in microwaved *D. maruadsi* surimi products (MIC). The intensity of detection of hexanal, pentanal, and 3-methylbutanal were most pronounced in FRI, followed by in STE. This is probably because FRI was heated at the highest temperature, which deepened the lipid oxidation, forming large amounts of aldehydes [42]. At the same time, the addition of TGase provided more free amino acids as precursors [61], and the intensity of the aldehydes of FRI in NT justifies the above speculation.

The detection results of alcohols in NT and WT were more similar, with 11 and 10 species, respectively. 1-butanol, 1-propanol, ethanol, isopentanol, and isobutanol were detected with high intensity, and all were strongly represented in the MIC and relatively weak in the FRI. This may be due to the high temperature setting of the FRI (150 °C), which promoted the esterification reaction, while the MIC did the opposite. This result is similar to the findings of Luo et al. [57]. In addition, 3-octanol, 1-octen-3-ol, 1-hexanol, 2-methyl-1-butanol, and isobutanol were detected in the individual samples. Alcohols can be formed by lipid oxidation, amino acid reduction, and carbohydrate metabolism [63]. Generally, due to the high threshold of alcohols, its influence on the overall flavor of surimi products is small [64]. However, 1-octen-3-ol has a distinct mushroom and earthy odor and is the main flavor characteristic substance of surimi products [65]. Although it was detected in all samples in this study, the intensity was slightly lower. This may be because the earthy smell of 1-octen-3-ol is more likely to be formed in freshwater surimi products, but less in *D. maruadsi* (sea fish). A study by Yi et al. found that the content of 1-octen-3-ol decreased as the proportion of seawater surimi (anchovy) in the fresh/seawater mix surimi decreased [63].

The types of ketone volatile compounds in NT and WT were relatively similar, detected as 10 and 11, respectively. Among them, 2-pentanone, 2-butanone, 2-propanone, 2,3-pentanedione, and acetoin were detected with higher intensities, and they had the odor of cheese and butter. These ketone volatiles were also derived from lipid oxidation and amino acid catabolism. The ketones were detected at similar intensities in BOI and STE and at intermediate levels in the samples of each treatment group, while they were detected at higher intensities in MIC. This is due to the non-thermal effect of microwaves, which expose more hydrophobic and sulfhydryl binding sites, and the ability of the treated myogenic fibrils to bind ketone flavor compounds was higher than the other treatment groups [66].

Esters in NT and WT were detected as four and five, respectively. All contained butyl butanoate, butyl propanoate, butyl acetate, ethyl acetate. The esters usually have a sweet and typically fruity flavor and are the result of the esterification of acids and alcohols [63]. They had higher detectable intensities in FRI, especially ethyl acetate, but lower intensities in MIC. Given the low intensity of most of the alcohols detected in the FRI samples, represented by ethanol, and the higher intensity in the MIC. It can be concluded that the esterification reaction was more intense in FRI and weaker in MIC. This is related to the high-temperature treatment of FRI (150 °C) [67].

In NT and WT, the species and distribution of furans and pyrazines were consistent. 2-ethylfuran and 2-pentylfuran are considered to have a meaty and caramel flavor, and methylpyrazine has a barbecue flavor, both of which can be obtained by the Maillard reaction [57]. Their higher detection intensities in back-pressure sterilized *D. maruadsi* surimi products (BAC) may be due to the combination of the higher moisture content and the heat treatment temperature (110 °C) which facilitates the Maillard reaction [68]. This also resulted in higher detection intensity for all other types of volatile compounds in BAC. It is speculated that the darker chromaticity of BAC may be related to the Maillard reaction effect. Compared to BAC, roasted *D. maruadsi* surimi products (ROA) and MIC had lower heating temperatures and moisture content, as well as the lowest detection intensity of furans. This also further verified the determinative effect of Maillard reaction on furans in the sample.

**Figure 7:**
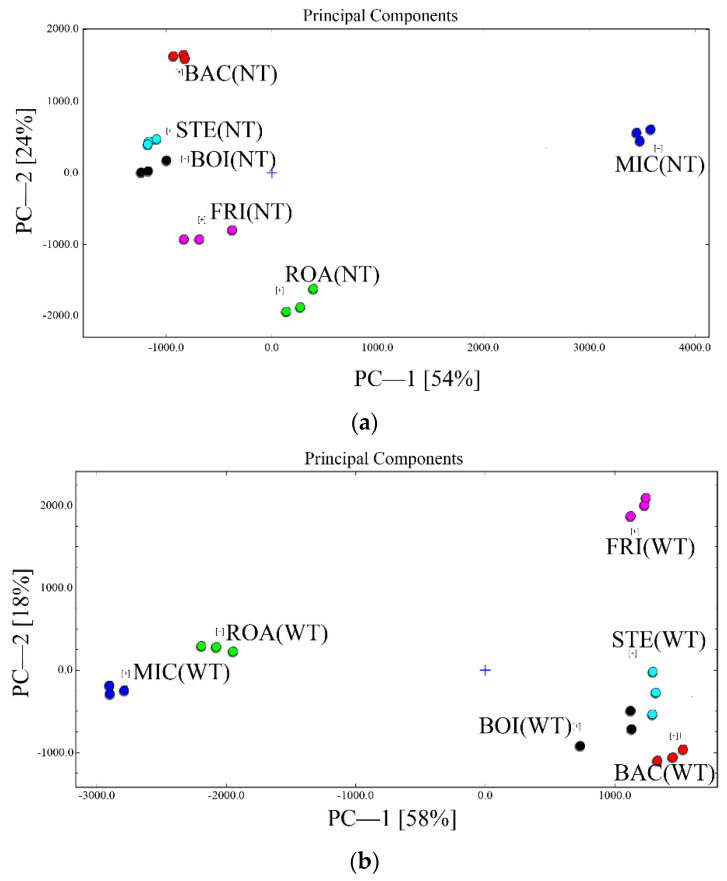
The principal component analysis of volatile flavor substances with surimi products subjected to different thermal treatment methods. (**a**): NT; (**b**): WT; BOI: boiled *D. maruadsi* surimi products; STE: steamed *D. maruadsi* surimi products; BAC: back-pressure sterilized *D. maruadsi* surimi products; ROA: roasted *D. maruadsi* surimi products; MIC: microwaved *D. maruadsi* surimi products; FRI: fried *D. maruadsi* surimi products; NT: group of without TGase; WT: group of with TGase.

The results of the principal component analysis of the volatile compounds for each sample in NT and WT are shown in Figure 7. Overall, the aromatic characteristics of BOI and STE are similar, regardless of TGase addition. This is due to the similar heating patterns and temperatures of the BOI and STE. In contrast to BOI and STE, BAC, ROA, MIC, and FRI showed their own unique aromatic characteristics. Temperature was the main factor contributing to the differences in aroma between the treatment groups of samples. Zhang et al. [42] revealed that the heat-treatment temperature had a significant effect on the volatile components in surimi gels, which was further confirmed by the results of this study. In practice, however, this reaction is complex and is influenced by a number of factors. When studying the effect of water activity (a_w_) on the formation of volatile compounds from whey proteins during high-temperature storage, it was found that the formation of volatile compounds was enhanced at high a_w_ values [69]. Therefore, ROA, MIC, and FRI may result in lower a_w_ due to the evaporation and transfer of free water during the high temperature gel phase, which in turn affects the formation of their volatile flavor substances. MIC had the lowest gelation temperature among the six heat-treatment methods due to the characteristics of its heating principle and low moisture content. This ultimately results in the most pronounced flavor profile.

## 4. Conclusions

Different thermal treatment methods could change the gel characteristics of surimi products with or without TGase and provide different flavor characteristics. In boiled (BOI) and steamed (STE) *D. maruadsi* surimi products, their heat treatments and temperature settings were found to be more conducive to the full unfolding and re-agglomeration of protein structures in *D. maruadsi* surimi products. The ability to form important chemical forces such as disulphide bonds was equally strong among them. The result was a compact gel structure with aldehydes as the main flavor component. Compared to BOI and STE, the surimi products obtained by the other four thermal treatments all showed their own characteristics. Back-pressure sterilized *D. maruadsi* surimi products (BAC) had a short low-temperature gel time and the proteins aggregated rapidly without fully unfolding, resulting in a loose and poorly water-retaining gel structure. However, the high temperature of 110 °C promoted the occurrence of the Maillard reaction in the BAC, leading to more formation of the flavor components such as furan. Due to the limitations of processing characteristics, roasted (ROA), microwaved (MIC), and fried (FRI) *D. maruadsi* surimi products need to be heat treated in a relatively more open environment. This caused them to evaporate a lot of water during the heat treatment. Due to the special heating principle and low moisture content of MIC, the degree of gelation was insufficient, forming a loose gel structure with alcohols and ketones as the main flavor components. ROA and FRI form a dense and highly water-holding gel structure after high temperature gelation due to the increase in protein concentration. FRI prepared by high-temperature frying contains the most esterification products. The effect of TGase on the quality of *D. maruadsi* surimi products in each treatment group was consistent with most existing research results. However, its effect was different under each thermal treatment method. The gel properties of ROA were most clearly enhanced by TGase. Additionally, TGase promoted the conversion of more free amino acids to aldehydes in FRI. In summary, BOI and STE had better-quality gels and were richer in flavor components, both in terms of variety and content.

## Figures and Tables

**Table 1:** The process for preparing *D. maruadsi* surimi products using different thermal treatment methods.

Abbreviation	Group	Thermal Treatment Method
BOI	Boiled *D. maruadsi* surimi products	Firstly, in a constant temperature water bath at 40 °C for 30 min, and then at 90 °C for 20 min.
STE	Steamed *D. maruadsi* surimi products	Firstly, in a constant temperature water bath at 40 °C for 30 min, and then steam in a steamer with boiling water for 17 min.
BAC	Back-pressure sterilized *D. maruadsi* surimi products	Heated in a back pressure sterilizer at 110 °C and 0.05 MPa for 10 min.
ROA	Roasted *D. maruadsi* surimi products	Firstly, in a constant temperature water bath at 40 °C for 30 min, then removed the nylon casing, and finally roasted in an oven at 100 °C for 18 min.
MIC	Microwaved *D. maruadsi* surimi products	Firstly, in a constant temperature water bath at 40 °C for 30 min, then removed the casing, and finally heated it in a 2450 MHz microwave oven with a power of 3 W/g for 1 min. (The surface temperature was 72.4 ℃)
FRI	Fried *D. maruadsi* surimi products	Firstly, in a constant temperature water bath at 40 °C for 30 min, then removed the casing, and finally fried in a fryer at 150 °C for 3 min.

**Table 2:** Spectral peak areas of various water states in *D. maruadsi* surimi products.

Thermal Treatment Method	T_21_ + T_22_	T_2__3_	T_2__4_
	Samples without TGase (NT)		
BOI	19.76 ± 2.80 ^a^	954.77 ± 1.23 ^a^	52.04 ± 3.92 ^b^
STE	25.11 ± 4.23 ^a^	944.69 ± 4.03 ^a^	57.59 ± 1.85 ^ab^
BAC	28.84 ± 2.77 ^a^	915.71 ± 15.39 ^ab^	65.24 ± 1.96 ^a^
ROA	25.07 ± 5.31 ^a^	885.56 ± 8.02 ^bc^	34.87 ± 0.97 ^cd^
MIC	19.74 ± 0.44 ^a^	881.10 ± 24.57 ^bc^	37.31 ± 3.62 ^c^
FRI	29.69 ± 5.73 ^a^	851.85 ± 18.64 ^c^	27.66 ± 1.17 ^d^
	Samples with TGase (WT)		
BOI	23.44 ± 2.55 ^a^	1113.34 ± 28.79 ^a^	11.90 ± 1.93 ^b^^c^
STE	30.71 ± 2.34 ^a^	1057.69 ± 40.04 ^ab^	15.78 ± 2.56 ^b^
BAC	21.85 ± 6.87 ^a^	1018.18 ± 15.94 ^bc^	34.45 ± 4.10 ^a^
ROA	30.56 ± 5.57 ^a^	979.97 ± 8.36 ^c^	7.33 ± 0.38 ^cd^
MIC	27.34 ± 3.19 ^a^	962.03 ± 5.92 ^c^	9.31 ± 0.37 ^b^^cd^
FRI	25.17 ± 2.08 ^a^	954.91 ± 15.84 ^c^	3.79 ± 0.08 ^d^

Completely different letters in the same indicator indicate significant differences between the same columns (*p* < 0.05). BOI: boiled *D. maruadsi* surimi products; STE: steamed *D. maruadsi* surimi products; BAC: back-pressure sterilized *D. maruadsi* surimi products; ROA: roasted *D. maruadsi* surimi products; MIC: microwaved *D. maruadsi* surimi products; FRI: fried *D. maruadsi* surimi products.

**Table 3:** T_2_ relaxation peak areas of two *D. maruadsi* surimi products (NT and WT) under different thermal treatment methods.

	Moisture Content	T_21_ + T_22_	T_23_	T_24_
	Samples without TGase (NT)			
Water loss rate	0.856 *	−0.316	0.678	0.862 *
Moisture content	1	−0.384	0.916 *	0.900 *
	Samples with TGase (WT)			
Water loss rate	0.805	−0.270	0.420	0.834 *
Moisture content	1	−0.200	0.851 *	0.668

* Represents *p* < 0.05.

**Table 4:** Different thermal treatment methods and the addition of TGase affect the brightness and whiteness of *D. maruadsi* surimi products.

Thermal Treatment Method	a*	b*	L*	Whiteness
	Samples without TGase (NT)			
BOI	−5.07 ± 0.01 ^c^	12.54 ± 0.08 ^b^	74.40 ± 0.23 ^a^	71.05 ± 0.23 ^a^
STE	−5.03 ± 0.03 ^c^	12.58 ± 0.04 ^b^	74.5 ± 0.15 ^a^	71.12 ± 0.12 ^a^
BAC	−3.82 ± 0.03 ^a^	16.57 ± 0.04 ^a^	74.10 ± 0.08 ^a^	69.01 ± 0.24 ^c^
ROA	−5.07 ± 0.06 ^c^	11.91 ± 0.06 ^c^	73.21 ± 0.16 ^b^	70.25 ± 0.16 ^b^
MIC	−4.67 ± 0.02 ^b^	11.72 ± 0.15 ^c^	71.49 ± 0.24 ^c^	68.81 ± 0.21 ^c^
FRI	−5.26 ± 0.02 ^c^	12.23 ± 0.13 ^c^	73.23 ± 0.07 ^b^	70.10 ± 0.07 ^b^
	Samples with TGase (WT)			
BOI	−5.12 ± 0.01 ^c^	12.42 ± 0.07 ^b^	76.42 ± 0.17 ^a^	72.86 ± 0.17 ^a^
STE	−5.09 ± 0.01 ^c^	12.51 ± 0.02 ^b^	76.34 ± 0.26 ^a^	72.75 ± 0.23 ^a^
BAC	−3.91 ± 0.08 ^a^	16.68 ± 0.17 ^a^	74.81 ± 0.24 ^b^	69.53 ± 0.20 ^c^
ROA	−5.03 ± 0.05 ^c^	11.95 ± 0.05 ^c^	74.71 ± 0.25 ^b^	71.58 ± 0.21 ^b^
MIC	−4.42 ± 0.04 ^b^	11.45 ± 0.03 ^d^	72.77 ± 0.69 ^c^	70.13 ± 0.62 ^c^
FRI	−5.26 ± 0.02 ^d^	12.45 ± 0.05 ^b^	75.10 ± 0.17 ^b^	71.67 ± 0.15 ^b^

Completely different letters in the same indicator indicate significant differences between the same columns (*p* < 0.05). BOI: boiled *D. maruadsi* surimi products; STE: steamed *D. maruadsi* surimi products; BAC: back-pressure sterilized *D. maruadsi* surimi products; ROA: roasted *D. maruadsi* surimi products; MIC: microwaved *D. maruadsi* surimi products; FRI: fried *D. maruadsi* surimi products.

**Table 5:** The correlation analysis on fracture coefficient with chemical force and secondary structure of *D. maruadsi* surimi products under different thermal treatment methods.

		Group of without TGase (NT)	Group of with TGase (WT)
Gel strength	α-helix	−0.985 **	−0.992 **
	β-sheet	0.982 **	0.957 **
	β-turns	0.980 **	0.866 *
	Unordered	0.907 *	0.939 **
	Ionic bond	−0.252	−0.086
	Hydrogen bonds	−0.900 *	−0.892 *
	Hydrophobic interaction	0.867 *	0.972 **
	Disulfide bonds	0.432	0.539

* represents *p* < 0.05, and ** represents *p* < 0.01.

## Data Availability

Data is contained within the article or Supplementary Material.

## Supplementary Materials

The following are available online at https://www.mdpi.com/article/10.3390/foods11010066/s1, Table S1: The signal intensity of volatile compounds in *D. maruadsi* surimi products under different thermal treatment methods (NT), Table S2: The signal intensity of volatile compounds in *D. maruadsi* surimi products under different thermal treatment methods (WT).
