# Supplementary material for: Different Thermal Treatment Methods and TGase Addition Affect Gel Quality and Flavour Characteristics of Decapterus maruadsi Surimi Products
Source: Foods. 2021 Dec 28;11(1):66. doi: 10.3390/foods11010066 (PMC8750094; doi:10.3390/foods11010066)
Supplement: Supplementary file 1 [file foods-11-00066-s001.zip › foods-1495480-supplementary.pdf]

**Table S1.** The signal intensity of volatile compounds in *D. maruadsi* surimi products under different thermal treatment methods (NT).

| Compounds | RI | Rt [s] | Dt [RIPrel] | Peak intensity of surimi products (NT) | | | | | |
|---|---|---|---|---|---|---|---|---|---|
| | | | | BOI | STE | BAC | ROA | MIC | FRI |
| **Aldehydes (14)** | | | | | | | | | |
| Nonanal | 1111 | 509 | 1.47 | 675 ± 13$^{a}$ | 663 ± 2$^{a}$ | 560 ± 16$^{b}$ | 550 ± 36$^{b}$ | 263 ± 8$^{c}$ | 568 ± 21$^{b}$ |
| Octanal | 1006 | 359 | 1.41 | 444 ± 7$^{a}$ | 366 ± 8$^{b}$ | 366 ± 7$^{b}$ | 296 ± 22$^{c}$ | 152 ± 3$^{d}$ | 320 ± 10$^{c}$ |
| Benzaldehyde | 957 | 312 | 1.15 | 223 ± 6$^{b}$ | 179 ± 1$^{c}$ | 355 ± 9$^{a}$ | 125 ± 5$^{e}$ | 80 ± 5$^{f}$ | 151 ± 2$^{d}$ |
| (E)-2-Heptenal | 945 | 301 | 1.27 | 68 ± 2$^{a}$ | 57 ± 3$^{bc}$ | 58 ± 4$^{bc}$ | 39 ± 1$^{d}$ | 53 ± 1$^{c}$ | 62 ± 0$^{ab}$ |
| Heptanal-M | 901 | 264 | 1.33 | 989 ± 16$^{a}$ | 880 ± 3$^{b}$ | 704 ± 4$^{d}$ | 611 ± 30$^{e}$ | 324 ± 21$^{f}$ | 814 ± 15$^{c}$ |
| Heptanal-D | 901 | 264 | 1.70 | 308 ± 7$^{a}$ | 233 ± 1$^{b}$ | 151 ± 6$^{d}$ | 92 ± 7$^{e}$ | 44 ± 5$^{f}$ | 180 ± 5$^{c}$ |
| (E)-2-Hexenal | 845 | 232 | 1.18 | 143 ± 2$^{c}$ | 126 ± 0$^{cd}$ | 109 ± 1$^{d}$ | 186 ± 11$^{a}$ | 197 ± 9$^{a}$ | 164 ± 4$^{b}$ |
| Hexanal-M | 790 | 204 | 1.26 | 1085 ± 10$^{a}$ | 1057 ± 3$^{a}$ | 950 ± 2$^{b}$ | 967 ± 19$^{b}$ | 649 ± 38$^{c}$ | 1116 ± 17$^{a}$ |
| Hexanal-D | 790 | 203 | 1.57 | 1509 ± 15$^{a}$ | 1217 ± 6$^{b}$ | 949 ± 25$^{c}$ | 866 ± 43$^{c}$ | 135 ± 28$^{d}$ | 1229 ± 61$^{b}$ |
| (E)-2-Pentenal | 744 | 184 | 1.11 | 126 ± 1$^{b}$ | 101 ± 2$^{cd}$ | 98 ± 2$^{d}$ | 161 ± 7$^{a}$ | 67 ± 5$^{e}$ | 112 ± 5$^{c}$ |
| Pentanal-M | 693 | 163 | 1.18 | 430 ± 25$^{b}$ | 485 ± 10$^{b}$ | 332 ± 2$^{c}$ | 209 ± 10$^{d}$ | 61 ± 1$^{e}$ | 559 ± 45$^{a}$ |
| Pentanal-D | 692 | 163 | 1.43 | 143 ± 7$^{b}$ | 140 ± 4$^{b}$ | 91 ± 2$^{bc}$ | 97 ± 9$^{b}$ | 38 ± 1$^{c}$ | 306 ± 41$^{a}$ |
| 2-Methylbutanal-M | 657 | 153 | 1.16 | 606 ± 5$^{b}$ | 620 ± 7$^{ab}$ | 643 ± 3$^{a}$ | 356 ± 3$^{d}$ | 247 ± 3$^{e}$ | 550 ± 24$^{c}$ |
| 2-Methylbutanal-D | 657 | 153 | 1.40 | 1692 ± 6$^{a}$ | 1804 ± 7$^{a}$ | 1841 ± 26$^{a}$ | 804 ± 144$^{c}$ | 1774 ± 118$^{a}$ | 1147 ± 44$^{b}$ |
| 3-Methylbutanal-M | 636 | 148 | 1.17 | 856 ± 7$^{bc}$ | 876 ± 6$^{ab}$ | 789 ± 4$^{c}$ | 927 ± 46$^{ab}$ | 948 ± 30$^{a}$ | 927 ± 6$^{ab}$ |
| 3-Methylbutanal-D | 638 | 148 | 1.41 | 1103 ± 21$^{bc}$ | 1194 ± 15$^{b}$ | 998 ± 14$^{bc}$ | 630 ± 163$^{d}$ | 1956 ± 115$^{a}$ | 836 ± 36$^{cd}$ |
| Butanal | 586 | 134 | 1.29 | 581 ± 2$^{c}$ | 659 ± 2$^{b}$ | 901 ± 13$^{a}$ | 303 ± 13$^{e}$ | 158 ± 3$^{f}$ | 485 ± 9$^{d}$ |
| Methylpropanal | 548 | 125 | 1.28 | 112 ± 2$^{c}$ | 148 ± 2$^{b}$ | 243 ± 5$^{a}$ | 30 ± 3$^{e}$ | 51 ± 3$^{d}$ | 48 ± 3$^{d}$ |
| Benzeneacetaldehyde | 1039 | 406 | 1.25 | 76 ± 8$^{b}$ | 80 ± 4$^{b}$ | 80 ± 7$^{b}$ | 83 ± 6$^{b}$ | 300 ± 24$^{a}$ | 99 ± 1$^{b}$ |
| **Alcohols (11)** | | | | | | | | | |
| 3-Octanol | 989 | 338 | 1.40 | 186 ± 5$^{c}$ | 146 ± 3$^{d}$ | 130 ± 1$^{d}$ | 397 ± 22$^{a}$ | 278 ± 13$^{b}$ | 190 ± 5$^{c}$ |
| 1-Octen-3-ol | 984 | 334 | 1.17 | 295 ± 9$^{b}$ | 273 ± 4$^{d}$ | 248 ± 5$^{d}$ | 280 ± 10$^{bc}$ | 409 ± 9$^{a}$ | 255 ± 9$^{cd}$ |
| 1-Hexanol-M | 870 | 245 | 1.33 | 510 ± 14$^{a}$ | 486 ± 10$^{a}$ | 420 ± 7$^{bc}$ | 496 ± 15$^{a}$ | 382 ± 19$^{c}$ | 427 ± 5$^{b}$ |
| 1-Hexanol-D | 868 | 244 | 1.63 | 61 ± 1$^{a}$ | 51 ± 1$^{bc}$ | 51 ± 2$^{bc}$ | 55 ± 1$^{b}$ | 47 ± 2$^{cd}$ | 45 ± 2$^{d}$ |
| 2-Furanmethanol | 850 | 235 | 1.12 | 133 ± 6$^{a}$ | 111 ± 4$^{b}$ | 95 ± 3$^{c}$ | 87 ± 3$^{c}$ | 68 ± 3$^{d}$ | 71 ± 1$^{d}$ |
| 1-Pentanol | 759 | 190 | 1.25 | 211 ± 7$^{c}$ | 192 ± 1$^{c}$ | 224 ± 1$^{c}$ | 223 ± 10$^{c}$ | 537 ± 25$^{a}$ | 475 ± 25$^{b}$ |
| 2-Methyl-1-butanol | 730 | 179 | 1.23 | 84 ± 5$^{a}$ | 91 ± 2$^{a}$ | 81 ± 1$^{a}$ | 66 ± 5$^{b}$ | 51 ± 3$^{c}$ | 62 ± 4$^{b}$ |
| 1-Butanol | 691 | 162 | 1.36 | 515 ± 7$^{b}$ | 342 ± 6$^{d}$ | 420 ± 4$^{c}$ | 282 ± 7$^{e}$ | 1010 ± 39$^{a}$ | 282 ± 1$^{e}$ |
| 1-Propanol | 538 | 122 | 1.11 | 1458 ± 12$^{b}$ | 1541 ± 14$^{b}$ | 1465 ± 13$^{b}$ | 1266 ± 49$^{c}$ | 1635 ± 45$^{a}$ | 1181 ± 16$^{c}$ |
| Ethanol | 449 | 98 | 1.05 | 542 ± 20$^{bc}$ | 509 ± 1$^{bc}$ | 490 ± 5$^{bc}$ | 586 ± 21$^{b}$ | 1457 ± 73$^{a}$ | 474 ± 15$^{c}$ |
| Isopentanol | 728 | 178 | 1.50 | 96 ± 3$^{b}$ | 112 ± 5$^{b}$ | 102 ± 1$^{b}$ | 105 ± 16$^{b}$ | 2021 ± 187$^{a}$ | 110 ± 2$^{b}$ |
| Isobutanol | 605 | 140 | 1.17 | 160 ± 2$^{c}$ | 171 ± 9$^{c}$ | 174 ± 3$^{c}$ | 215 ± 12$^{b}$ | 949 ± 24$^{a}$ | 101 ± 4$^{d}$ |
| **Ketones (10)** | | | | | | | | | |
| Cyclohexanone | 899 | 263 | 1.15 | 545 ± 10$^{b}$ | 601 ± 1$^{a}$ | 544 ± 10$^{b}$ | 357 ± 14$^{c}$ | 200 ± 11$^{d}$ | 344 ± 11$^{c}$ |
| 2-Heptanone | 891 | 256 | 1.26 | 146 ± 1$^{d}$ | 167 ± 2$^{c}$ | 356 ± 3$^{a}$ | 104 ± 3$^{e}$ | 299 ± 8$^{b}$ | 144 ± 3$^{d}$ |
| 2-Hexanone | 780 | 199 | 1.18 | 207 ± 4$^{c}$ | 257 ± 3$^{b}$ | 268 ± 3$^{a}$ | 112 ± 1$^{e}$ | 68 ± 2$^{f}$ | 162 ± 4$^{d}$ |
| 3-Pentanone | 692 | 163 | 1.11 | 293 ± 8$^{b}$ | 244 ± 5$^{c}$ | 248 ± 1$^{c}$ | 208 ± 7$^{d}$ | 321 ± 7$^{a}$ | 176 ± 2$^{e}$ |
| 2-Pentanone | 674 | 158 | 1.37 | 412 ± 9$^{c}$ | 396 ± 10$^{c}$ | 494 ± 8$^{b}$ | 403 ± 11$^{c}$ | 711 ± 1$^{a}$ | 291 ± 4$^{d}$ |
| 2-Butanone | 566 | 129 | 1.25 | 414 ± 5$^{c}$ | 605 ± 19$^{b}$ | 1536 ± 20$^{a}$ | 215 ± 15$^{f}$ | 345 ± 6$^{d}$ | 273 ± 7$^{e}$ |

**Table S1. Continued**

| Compounds | RI | Rt [s] | Dt [RIPrel] | Peak intensity of surimi products (NT) | | | | | |
|---|---|---|---|---|---|---|---|---|---|
| | | | | BOI | STE | BAC | ROA | MIC | FRI |
| 2-Propanone | 495 | 110 | 1.12 | 416 ± 18$^{cd}$ | 702 ± 27$^{b}$ | 2543 ± 109$^{a}$ | 284 ± 21$^{d}$ | 497 ± 43$^{c}$ | 330 ± 18$^{d}$ |
| 2,3-Pentanedione | 690 | 162 | 1.21 | 243 ± 15$^{d}$ | 227 ± 3$^{d}$ | 223 ± 3$^{d}$ | 1004 ± 6$^{a}$ | 288 ± 18$^{c}$ | 381 ± 1$^{b}$ |
| Acetoin-M | 709 | 170 | 1.06 | 510 ± 184$^{b}$ | 297 ± 6$^{b}$ | 409 ± 11$^{b}$ | 578 ± 159$^{b}$ | 1033 ± 40$^{a}$ | 630 ± 223$^{ab}$ |
| Acetoin-D | 709 | 170 | 1.33 | 187 ± 77$^{b}$ | 89 ± 0$^{b}$ | 123 ± 5$^{b}$ | 219 ± 79$^{b}$ | 2674 ± 300$^{a}$ | 257 ± 107$^{b}$ |
| 6-Methyl-5-hepten-2-one | 990 | 339 | 1.19 | 180 ± 3$^{c}$ | 185 ± 5$^{c}$ | 201 ± 4$^{b}$ | 156 ± 7$^{d}$ | 234 ± 4$^{a}$ | 142 ± 4$^{d}$ |
| **Esters (4)** | | | | | | | | | |
| Butylbutanoate | 1003 | 355 | 1.34 | 204 ± 7$^{a}$ | 140 ± 1$^{c}$ | 171 ± 4$^{b}$ | 98 ± 7$^{d}$ | 71 ± 2$^{e}$ | 132 ± 2$^{c}$ |
| Butylpropanoate | 923 | 283 | 1.29 | 57 ± 2$^{a}$ | 39 ± 1$^{c}$ | 48 ± 3$^{b}$ | 31 ± 1$^{d}$ | 23 ± 2$^{e}$ | 39 ± 3$^{c}$ |
| Butylacetate | 802 | 210 | 1.24 | 44 ± 2$^{c}$ | 45 ± 3$^{c}$ | 37 ± 3$^{c}$ | 16 ± 1$^{d}$ | 146 ± 8$^{a}$ | 66 ± 3$^{b}$ |
| EthylAcetate-M | 598 | 138 | 1.10 | 1130 ± 13$^{b}$ | 1049 ± 23$^{b}$ | 830 ± 11$^{c}$ | 856 ± 80$^{c}$ | 180 ± 8$^{d}$ | 1418 ± 5$^{a}$ |
| EthylAcetate-D | 597 | 137 | 1.34 | 1496 ± 21$^{b}$ | 1278 ± 53$^{c}$ | 988 ± 25$^{d}$ | 661 ± 115$^{e}$ | 332 ± 20$^{f}$ | 2895 ± 20$^{a}$ |
| **Furans and Pyrazines (3)** | | | | | | | | | |
| 2-Ethylfuran | 694 | 164 | 1.05 | 101 ± 8$^{c}$ | 151 ± 1$^{b}$ | 445 ± 15$^{a}$ | 73 ± 10$^{c}$ | 91 ± 3$^{c}$ | 84 ± 8$^{c}$ |
| 2-Pentylfuran | 995 | 344 | 1.26 | 20 ± 2$^{b}$ | 20 ± 2$^{b}$ | 42 ± 3$^{a}$ | 18 ± 3$^{b}$ | 23 ± 1$^{b}$ | 39 ± 4$^{a}$ |
| Methylpyrazine | 796 | 206 | 1.07 | 85 ± 2$^{d}$ | 75 ± 3$^{d}$ | 139 ± 2$^{b}$ | 231 ± 9$^{a}$ | 111 ± 7$^{c}$ | 110 ± 5$^{c}$ |
| **Acids (1)** | | | | | | | | | |
| Aceticacid | 617 | 143 | 1.07 | 202 ± 2$^{d}$ | 219 ± 3$^{d}$ | 253 ± 8$^{c}$ | 445 ± 5$^{a}$ | 312 ± 8$^{b}$ | 302 ± 4$^{b}$ |
| **Other (1)** | | | | | | | | | |
| Toluene | 767 | 194 | 1.02 | 193 ± 6$^{a}$ | 159 ± 4$^{b}$ | 145 ± 6$^{b}$ | 199 ± 4$^{a}$ | 184 ± 6$^{a}$ | 146 ± 5$^{b}$ |

RI: Retention index; Rt: Retention time in the capillary GC column; Dt: The drift time in the drift tube; BOI: Boiled *D.maruadsi* surimi products; STE: Steamed *D.maruadsi* surimi products; BAC: Back-pressure sterilized *D.maruadsi* surimi products; ROA: Roasted *D.maruadsi* surimi products; MIC: Microwaved *D.maruadsi* surimi products; FRI: Fried *D.maruadsi* surimi products; NT: group of without TGase; WT: group of with TGase.

**Table S2.** The signal intensity of volatile compounds in *D. maruadsi* surimi products under different thermal treatment methods (WT).

| Compounds | RI | Rt [s] | Dt [RIPrel] | Peak intensity of surimi products (WT) | | | | | |
|---|---|---|---|---|---|---|---|---|---|
| | | | | BOI | STE | BAC | ROA | MIC | FRI |
| **Aldehydes (16)** | | | | | | | | | |
| Nonanal | 1111 | 509 | 1.47 | 1039 ± 27[a] | 838 ± 48[b] | 656 ± 2[c] | 468 ± 10[d] | 327 ± 9[e] | 661 ± 18[c] |
| Octanal | 1006 | 359 | 1.41 | 523 ± 12[a] | 479 ± 29[b] | 409 ± 6[c] | 253 ± 4[e] | 160 ± 5[f] | 364 ± 11[d] |
| Benzaldehyde | 957 | 312 | 1.15 | 413 ± 23[a] | 322 ± 22[b] | 386 ± 12[a] | 136 ± 5[d] | 94 ± 5[d] | 225 ± 4[c] |
| (E)-2-Heptenal | 945 | 301 | 1.27 | 70 ± 2[a] | 71 ± 2[a] | 68 ± 3[a] | 49 ± 3[b] | 47 ± 2[b] | 70 ± 2[a] |
| Heptanal-M | 901 | 264 | 1.33 | 906 ± 47[a] | 925 ± 21[a] | 724 ± 7[b] | 494 ± 13[c] | 350 ± 2[d] | 877 ± 7[a] |
| Heptanal-D | 901 | 264 | 1.70 | 375 ± 20[a] | 317 ± 28[b] | 181 ± 2[d] | 65 ± 2[e] | 46 ± 3[e] | 241 ± 1[c] |
| (E)-2-Hexenal | 845 | 232 | 1.18 | 97 ± 17[d] | 125 ± 4[c] | 113 ± 3[cd] | 236 ± 3[a] | 204 ± 5[b] | 180 ± 1[b] |
| Hexanal-M | 790 | 204 | 1.26 | 887 ± 48[cd] | 1006 ± 6[b] | 929 ± 7[c] | 819 ± 26[d] | 619 ± 22[e] | 1101 ± 4[a] |
| Hexanal-D | 790 | 203 | 1.57 | 1641 ± 117[a] | 1557 ± 84[a] | 1113 ± 11[b] | 599 ± 51[c] | 218 ± 14[d] | 1690 ± 3[a] |
| (E)-2-Pentenal | 744 | 184 | 1.11 | 145 ± 1[a] | 117 ± 5[b] | 89 ± 2[c] | 159 ± 7[a] | 129 ± 6[b] | 131 ± 4[b] |
| Pentanal-M | 693 | 163 | 1.18 | 374 ± 24[c] | 447 ± 5[b] | 336 ± 4[d] | 128 ± 7[e] | 77 ± 3[f] | 662 ± 9[a] |
| Pentanal-D | 692 | 163 | 1.43 | 204 ± 16[b] | 210 ± 18[b] | 135 ± 0[c] | 85 ± 4[d] | 44 ± 1[e] | 612 ± 16[a] |
| 2-Methylbutanal-M | 657 | 153 | 1.16 | 420 ± 20[c] | 515 ± 24[b] | 610 ± 7[a] | 334 ± 5[d] | 323 ± 3[d] | 579 ± 13[a] |
| 2-Methylbutanal-D | 657 | 153 | 1.40 | 1376 ± 36[d] | 1633 ± 60[c] | 1764 ± 19[b] | 1724 ± 20[bc] | 2168 ± 26[a] | 1737 ± 19[bc] |
| 3-Methylbutanal-M | 636 | 148 | 1.17 | 488 ± 33[c] | 654 ± 7[b] | 644 ± 17[b] | 787 ± 19[a] | 812 ± 1[a] | 819 ± 7[a] |
| 3-Methylbutanal-D | 638 | 148 | 1.41 | 629 ± 30[e] | 808 ± 31[d] | 771 ± 9[d] | 1513 ± 40[b] | 1820 ± 44[a] | 936 ± 19[b] |
| Butanal | 586 | 134 | 1.29 | 571 ± 8[c] | 733 ± 27[b] | 971 ± 5[a] | 334 ± 6[d] | 210 ± 14[e] | 609 ± 8[b] |
| Methylpropanal | 548 | 125 | 1.28 | 91 ± 1[c] | 124 ± 16[b] | 169 ± 4[a] | 69 ± 1[c] | 77 ± 7[c] | 114 ± 3[b] |
| Benzeneacetaldehyde | 1039 | 406 | 1.25 | 95 ± 10[c] | 76 ± 2[c] | 82 ± 7[c] | 132 ± 11[b] | 161 ± 10[a] | 97 ± 3[c] |
| Decanal | 1274 | 743 | 1.53 | 377 ± 30[a] | 366 ± 24[a] | 354 ± 42[a] | 311 ± 7[a] | 315 ± 12[a] | 333 ± 8[a] |
| Furfural | 827 | 223 | 1.08 | 23 ± 1[cd] | 26 ± 3[c] | 42 ± 3[b] | 18 ± 0[d] | 20 ± 2[cd] | 48 ± 2[a] |
| **Alcohols (10)** | | | | | | | | | |
| 3-Octanol | 989 | 338 | 1.40 | 176 ± 20[c] | 160 ± 10[cd] | 129 ± 8[d] | 408 ± 6[a] | 299 ± 8[b] | 194 ± 6[c] |
| 1-Octen-3-ol | 984 | 334 | 1.17 | 490 ± 30[a] | 390 ± 11[cd] | 333 ± 3[d] | 440 ± 4[b] | 402 ± 3[bc] | 299 ± 3[d] |
| 1-Hexanol-M | 870 | 245 | 1.33 | 376 ± 17[c] | 423 ± 5[a] | 337 ± 6[d] | 412 ± 13[a] | 364 ± 5[d] | 399 ± 7[ab] |
| 1-Hexanol-D | 868 | 244 | 1.63 | 45 ± 1[a] | 51 ± 2[a] | 47 ± 5[a] | 47 ± 4[a] | 47 ± 3[a] | 50 ± 3[a] |
| 1-Pentanol | 759 | 190 | 1.25 | 174 ± 12[e] | 210 ± 2[d] | 218 ± 2[d] | 308 ± 7[c] | 362 ± 21[b] | 510 ± 10[a] |
| 2-Methyl-1-butanol | 730 | 179 | 1.23 | 105 ± 3[a] | 107 ± 4[a] | 77 ± 2[b] | 61 ± 7[c] | 84 ± 7[b] | 86 ± 1[b] |
| 1-Butanol | 691 | 162 | 1.36 | 532 ± 17[b] | 408 ± 17[c] | 409 ± 10[c] | 567 ± 15[b] | 737 ± 22[a] | 330 ± 5[d] |
| 1-Propanol | 538 | 122 | 1.11 | 1192 ± 44[d] | 1456 ± 8[b] | 1335 ± 18[c] | 1741 ± 12[a] | 1778 ± 15[a] | 1433 ± 26[b] |
| Ethanol | 449 | 98 | 1.05 | 662 ± 23[c] | 661 ± 17[c] | 591 ± 9[d] | 731 ± 33[b] | 1486 ± 12[a] | 605 ± 14[cd] |
| Isopentanol | 728 | 178 | 1.50 | 134 ± 5[c] | 145 ± 9[c] | 118 ± 12[c] | 528 ± 39[b] | 1081 ± 117[a] | 119 ± 4[c] |
| Isobutanol | 605 | 140 | 1.17 | 119 ± 19[c] | 102 ± 4[c] | 92 ± 2[c] | 297 ± 11[b] | 523 ± 24[a] | 81 ± 2[c] |
| **Ketones (11)** | | | | | | | | | |
| Cyclohexanone | 899 | 263 | 1.15 | 774 ± 9[a] | 760 ± 27[a] | 673 ± 2[b] | 381 ± 13[d] | 271 ± 4[e] | 451 ± 8[c] |
| 2-Heptanone | 891 | 256 | 1.26 | 146 ± 5[c] | 183 ± 4[b] | 396 ± 2[a] | 136 ± 1[c] | 194 ± 15[b] | 177 ± 1[b] |
| 2-Hexanone | 780 | 199 | 1.18 | 201 ± 12[b] | 272 ± 3[a] | 287 ± 1[a] | 101 ± 5[d] | 118 ± 5[d] | 177 ± 0[c] |
| 3-Pentanone | 692 | 163 | 1.11 | 228 ± 12[b] | 192 ± 2[c] | 194 ± 6[c] | 198 ± 7[c] | 274 ± 3[a] | 134 ± 1[d] |
| 2-Pentanone | 674 | 158 | 1.37 | 432 ± 12[c] | 441 ± 14[c] | 496 ± 6[b] | 512 ± 6[b] | 560 ± 3[a] | 304 ± 7[d] |

**Table S2. Continued**

| Compounds | RI | Rt [s] | Dt [RIPrel] | Peak intensity of surimi products (WT) | | | | | |
|---|---|---|---|---|---|---|---|---|---|
| | | | | BOI | STE | BAC | ROA | MIC | FRI |
| 2-Butanone | 566 | 129 | 1.25 | 373 ± 6[c] | 548 ± 36[b] | 1285 ± 26[a] | 210 ± 11[d] | 263 ± 5[d] | 374 ± 7[c] |
| 2-Propanone | 495 | 110 | 1.12 | 456 ± 45[cd] | 678 ± 31[b] | 2011 ± 29[a] | 411 ± 35[d] | 523 ± 19[c] | 522 ± 20[c] |
| 2,3-Pentanedione | 690 | 162 | 1.21 | 152 ± 3[d] | 134 ± 6[d] | 157 ± 5[d] | 604 ± 16[a] | 393 ± 9[b] | 268 ± 11[c] |
| Acetoin-M | 709 | 170 | 1.06 | 1350 ± 133[a] | 945 ± 83[b] | 837 ± 24[bc] | 1428 ± 42[a] | 1401 ± 20[a] | 592 ± 120[c] |
| Acetoin-D | 709 | 170 | 1.33 | 150 ± 10[b] | 126 ± 3[b] | 141 ± 5[b] | 1993 ± 548[a] | 2429 ± 230[a] | 185 ± 38[b] |
| 6-Methyl-5-hepten-2-one | 990 | 339 | 1.19 | 307 ± 6[ab] | 315 ± 9[ab] | 320 ± 1[a] | 285 ± 3[b] | 288 ± 19[ab] | 229 ± 8[c] |
| 3-Octanone | 991 | 341 | 1.30 | 152 ± 6[a] | 123 ± 5[b] | 116 ± 5[b] | 99 ± 1[c] | 99 ± 3[c] | 95 ± 2[c] |
| **Esters (5)** | | | | | | | | | |
| Butylbutanoate | 1003 | 355 | 1.34 | 215 ± 11[a] | 171 ± 6[b] | 183 ± 1[b] | 90 ± 5[d] | 65 ± 5[e] | 133 ± 3[c] |
| Butylpropanoate | 923 | 283 | 1.29 | 60 ± 2[a] | 54 ± 2[b] | 49 ± 1[c] | 26 ± 1[e] | 26 ± 0[e] | 39 ± 2[d] |
| EthylAcetate-M | 598 | 138 | 1.10 | 800 ± 52[d] | 1008 ± 34[b] | 931 ± 20[bc] | 835 ± 38[cd] | 429 ± 37[e] | 1340 ± 8[a] |
| EthylAcetate-D | 597 | 137 | 1.34 | 2406 ± 112[b] | 2456 ± 24[b] | 1706 ± 58[c] | 835 ± 61[d] | 347 ± 43[e] | 2815 ± 13[a] |
| Propylacetate | 705 | 168 | 1.16 | 108 ± 13[b] | 101 ± 9[b] | 106 ± 1[b] | 54 ± 4[c] | 66 ± 1[c] | 212 ± 8[a] |
| Butylacetate | 804 | 211 | 1.24 | 22 ± 4[a] | 13 ± 1[bc] | 10 ± 1[c] | 8 ± 1[c] | 18 ± 2[ab] | 24 ± 1[a] |
| **Furans and Pyrazines (3)** | | | | | | | | | |
| 2-Ethylfuran | 694 | 164 | 1.05 | 301 ± 24[b] | 288 ± 5[b] | 465 ± 9[a] | 130 ± 1[c] | 132 ± 4[c] | 103 ± 4[c] |
| 2-Pentylfuran | 995 | 344 | 1.26 | 25 ± 1[b] | 24 ± 1[b] | 46 ± 3[a] | 22 ± 0[b] | 20 ± 1[b] | 47 ± 3[a] |
| Methylpyrazine | 796 | 206 | 1.07 | 71 ± 7[e] | 75 ± 2[e] | 173 ± 5[b] | 202 ± 0[a] | 130 ± 1[c] | 110 ± 3[d] |
| **Acids (1)** | | | | | | | | | |
| Aceticacid | 617 | 143 | 1.07 | 110 ± 15[bc] | 110 ± 9[bc] | 101 ± 1[c] | 202 ± 4[a] | 188 ± 7[a] | 130 ± 2[b] |
| **Other (1)** | | | | | | | | | |
| Toluene | 767 | 194 | 1.02 | 207 ± 14[bc] | 192 ± 15[c] | 161 ± 2[d] | 241 ± 7[a] | 231 ± 5[ab] | 160 ± 3[d] |

RI: Retention index; Rt: Retention time in the capillary GC column; Dt: The drift time in the drift tube; BOI: Boiled *D.maruadsi* surimi products; STE: Steamed *D.maruadsi* surimi products; BAC: Back-pressure sterilized *D.maruadsi* surimi products; ROA: Roasted *D.maruadsi* surimi products; MIC: Microwaved *D.maruadsi* surimi products; FRI: Fried *D.maruadsi* surimi products; NT: group of without TGase; WT: group of with TGase.
